# Sclerosing epithelioid fibrosarcoma of the bone: a systematic review of radiologic findings and associated clinical features

**DOI:** 10.1097/MS9.0000000000005613

**Published:** 2026-09-08

**Authors:** Fatemeh Kanaani Nejad, Maryam Afshari, Alireza Keshtkar, Omid Bahrami, Amir H. Hoveidaei, Janet D. Conway

**Affiliations:** a Medical Imaging Research Center, Shiraz University of Medical Sciences, Shiraz, Iran; b Research Center for Noncommunicable Diseases, Jahrom University of Medical Sciences, Jahrom, Iran; c School of Medicine, Tehran University of Medical Sciences, Tehran, Iran; d Sports Medicine Research Center, Neuroscience Institute, Tehran University of Medical Sciences, Tehran, Iran; e International Center for Limb Lengthening, Rubin Institute for Advanced Orthopedics, Sinai Hospital of Baltimore, Baltimore, Maryland, USA

**Keywords:** bone neoplasm, cancer early diagnosis, diagnostic imaging, fibrosarcoma

## Abstract

**Background:**

Primary bone sclerosing epithelioid fibrosarcoma (SEF) is a rare, yet aggressive, condition associated with considerable mortality and prone to misdiagnosis or delayed diagnosis, making precise radiologic identification essential.

**Materials and methods:**

The systematic review was conducted in accordance with PRISMA standards. PubMed, Scopus, Web of Science, and Embase were searched through March 2025. The inclusion criteria consisted of English-language human studies with radiologic descriptions of primary bone SEF. Two independent reviewers conducted screening and data extraction. The risk of bias was evaluated utilizing the Joanna Briggs Institute critical appraisal tools for case reports and series.

**Results:**

Out of 190 identified papers, 18 met the inclusion criteria, describing 26 patients. The predominant findings in X-rays and computed tomography scans were lysis and destruction (88.46%), well-defined margins (81.81%), and soft tissue involvement (53.84%). magnetic resonance imaging (MRI) data revealed T1-weighted images exhibiting hypo- to isointense signals, while mixed hyper- and hypointense signals mostly characterized T2 sequences. SEF predominantly affected irregular bones (46.2%), followed by long bones (38.5%), with sclerotic rims being most prevalent in long bones (60%). Bone SEF predominantly manifested as pain alone in 40% of cases, and only 33.3% of the patients were initially classified as SEF. Accurate initial diagnosis of SEF was more frequently reported in studies that performed MRI.

**Conclusion:**

This systematic review elucidates the varied imaging features of bone SEF across several bone types. When these characteristics elicit suspicion in primary imaging, it is probably wise to prioritize MRI, and early biopsy contemplated to guarantee a timely and precise diagnosis.

## Introduction

Sclerosing epithelioid fibrosarcoma (SEF) is a rare and aggressive variant of soft tissue sarcoma initially characterized by Meis-Kindblom *et al* in 1995[1]. The typical histological characteristics are cords and sheets of epithelioid tumor cells within a dense collagenous extracellular matrix[1]. The condition has been documented to occur in various sites, predominantly within the deep soft tissue of the limbs and trunk, affecting skeletal muscle, fascia, and periosteum^[^2,3^]^. Recently, some cases of primary bone SEF have been reported, located in the flat or irregular bones of the craniofacial region, spine, or long bones^[^3–7^]^. Nonetheless, there is no agreement regarding the most common primary bone location.

HIGHLIGHTS

- SEF of bone primarily involves irregular bones, followed by long bones.
- Only 33% of cases of bone SEF were diagnosed correctly at first.
- Common imaging features include lysis, well-defined margins, and soft-tissue involvement.
- MRI revealed T1 hypo- to isointense and T2 mixed hyper- to hypointense signals.
- MRI-based studies showed higher diagnostic accuracy, indicating their clinical value.

Accurate and prompt detection of the condition is crucial because of its significant mortality, reported to be between 25% and 57%, as well as the high incidence of local recurrence and distant metastases, which occur in nearly half of patients^[^8–11^]^. Due to the rarity of the condition and its nonspecific clinical signs and symptoms, it may be easily misdiagnosed as benign conditions, such as granuloma or chondroblastoma^[^6,12^]^. Delayed diagnosis presents another significant challenge. According to a review, the interval from the first symptom presentation to diagnosis ranged from 1 month to 13 years, with a mean of 33 months[13]. Although its histopathological features are extensively characterized, the imaging characteristics across various bone types remain poorly understood, making accurate and timely diagnosis even more challenging.

Radiologically, bone SEF exhibits variability, potentially manifesting as a well-circumscribed tumor localized to bone or as a substantially destructive lesion with soft-tissue extension^[^5–7,13,14^]^. Imaging modalities, including X-ray, computed tomography (CT), magnetic resonance imaging (MRI), and positron emission tomography (PET), have been used in the literature for diagnosis, staging, and therapy planning. Nevertheless, numerous inconsistencies exist in the reported imaging characteristics^[^5,14–16^]^.

Given the absence of a comprehensive synthesis of imaging findings for SEF of bone, this systematic review aims to identify the imaging modalities most commonly employed in the diagnosis of SEF, summarize their reported findings, categorize these findings by bone type, and examine their clinical presentations and primary diagnoses.

## Materials and methods

This systematic review was conducted in accordance with the Preferred Reporting Items for Systematic Reviews and Meta-Analyses (PRISMA) guidelines[17] and was assessed with the AMSTAR (assessing the methodological quality of systematic reviews) guideline[18]. No artificial intelligence (AI) tools were used in the research or manuscript development; reporting is based on the TITAN Guidelines 2025 (Transparency in the Reporting of Artificial Intelligence) – governing the declaration and use of AI[19]. The review protocol was prospectively registered.

### Search strategy

A comprehensive systematic search was performed across four electronic medical databases, including PubMed, Scopus, Web of Science (WOS), and Embase, from their inception through March 2025 to identify relevant studies. The search used the following terms and relevant Medical Subject Headings (MeSH): “*sclerosing epithelioid fibrosarcoma,”* “*bone,”* “*bone tissue,”* “*bony apophysis,”* and “*condyle.”* The complete search strategy is provided in Supplemental Digital Content Table S1, available at: https://links.lww.com/MS9/B372. Additional articles were screened for eligibility by reviewing the reference lists of the included studies.

### Inclusion and exclusion criteria

This systematic review included studies that report imaging findings of SEF involving any type of bone (long, short, flat, or irregular), studies that report at least one imaging modality (e.g., X-ray, CT, MRI, or PET), human studies with no age or sex restrictions, and articles published in English with no date limitations. Studies were excluded if they focused solely on soft tissue SEF without primary bone involvement, lacked imaging data or descriptions, involved animals, in vitro experiments, or other experimental models, or were reviews, editorials, or opinion pieces without original data. Duplicate publications or overlapping patient cohorts were also excluded, with only the most comprehensive report retained.

### Screening and data extraction

FKN and MA independently screened the studies to identify suitable papers for inclusion in this study using Rayyan software, a free online tool for systematic reviews. OB and FKN extracted data using a pre-designed form that contained study information, patient age and sex, anatomical location of SEF, imaging findings, and patient outcomes. In case studies with multiple cases or in case series, all patients whose radiologic findings were described were analyzed. AHH resolved any disagreements between authors at each step.

### Outcomes and data synthesis

Radiological characteristics of the lesions were extracted from X-ray, CT, MRI, and PET imaging. These features were organized by the anatomical site of involvement, including the type of bone (long, short, flat, or irregular), and were analyzed alongside associated clinical presentations and pathological findings. Pathology was classified as either pure SEF (tumors exhibiting only the characteristic histopathological features of SEF) or hybrid SEF (tumors showing a mixture of SEF and low-grade fibromyxoid sarcoma (LGFMS) features). For individual cases, quantitative data were summarized as mean ± standard deviation (SD), while qualitative data were reported as frequencies and percentages.

### Risk of bias assessment

Quality assessment was conducted independently by OB and FKN. Since all included studies were either case series or case reports, the Joanna Briggs Institute (JBI) Critical Appraisal Checklists for case series and case reports were applied. The JBI checklists consist of ten questions for case series and eight questions for case reports.

### Study and patient characteristics

A total of 190 articles were identified through the search; after removal of 104 duplicate records, 86 articles were screened by title and abstract. After the removal of 21 records at this screening stage and 49 at the full-text screening stage, a total of 16 studies were included through the database search. Additionally, two articles were identified through reference list screening^[^20,21^]^. Ultimately, 18 articles, comprising four case series^[^3,5,6,22^]^ and 14 case reports^[^10,12–15,20,21,23–29^]^ were included in this study (Fig. 1). The results of the quality assessment for the included studies are provided in Supplemental Digital Content Tables S2 and S3, available at: https://links.lww.com/MS9/B372. In total, 26 patients were included, with a mean age of 34.76 ± 18.97 years. Fifteen patients (57.7%) were female, and the mean largest tumor diameter was 54.3 ± 21.4 mm.

**Figure 1.**
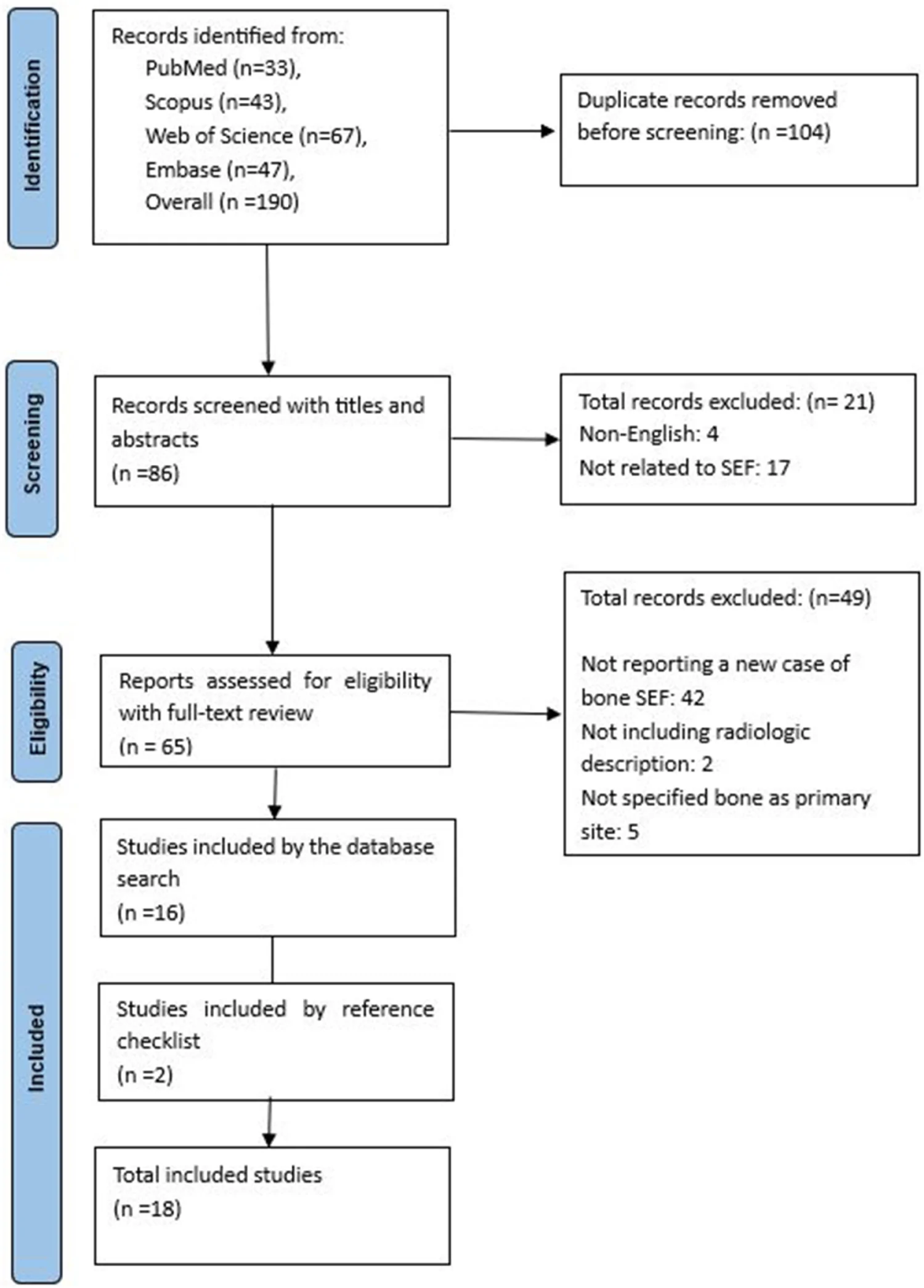
PRISMA flow diagram.

## Results

### Clinical presentations and primary diagnoses

Primary clinical presentations were reported in 20 cases. Of these, eight patients (40%) presented with pain alone, three patients (15%) with swelling alone, and five patients (25%) with both pain and swelling. Less common presentations included impaired weight-bearing with pain in one case (5%), limited mobility with pain in one case (5%), and tooth mobility with swelling in one case (5%).

The primary diagnosis was reported in 21 cases, of which seven (33.3%) were initially identified as SEF. Among the remaining 14 cases, three were initially diagnosed with benign conditions, including reactive fibrosis, chondroblastoma, and granuloma. Regarding pathological features, pure SEF morphology was observed in 21 cases (80.6%), while the remaining cases demonstrated hybrid pathological features. Detailed characteristics of the included cases are summarized in Table 1.

**Table 1:** Characteristics of the included studies and cases.

Author, year	Age	Sex	Performed imaging modalities	Location	Largest diameter (mm)	Initial diagnosis
J. Allen, 2021	49	M	CT	Mandible	30	Reactive fibrosis
L.T.C. Chow, 2004	48	F	X-ray, CT, PET	Sacrum	80	SEF
Q. Ding, 2020	19	M	CT, MRI	Fibula	32	SEF
G.S. Folk, 2007	22	F	CT	Mandible	57	Desmoplastic fibroma/ Osteosarcoma
T.G.P. Grunewald, 2010	16	F	X-ray, MRI	Tibia	53	Desmoplastic/non-ossifying fibroma
S. Kilaikodea, 2013	13	M	X-ray	Skull	25	Langerhans cell histiocytosis
K. Kosemehmetoglu, 2020	23	M	X-ray	Mandible	53	Not specified
K. Kosemehmetoglu, 2020	36	M	X-ray	Rib	70	Not specified
K. Kosemehmetoglu, 2020	71	M	X-ray	Clavicle	N/A	Not specified
K. Kosemehmetoglu, 2020	15	F	X-ray	Femur	77	Not specified
C. Ossendorf, 2008	32	F	X-ray, MRI	Ulna	50	SEF
A. Shenoy, 2019	16	F	CT	Mandible	N/A	Ewing sarcoma/ Osteosarcoma
D.I. Suster, 2024	26	F	X-ray, MRI	Femur	42	SEF
D.I. Suster, 2024	16	M	X-ray, CT	Clavicle	65	Low-grade fibromyxoid sarcoma
D.I. Suster, 2024	47	F	MRI	Skull	25	SEF
Y. Tsuda, 2019	23	F	X-ray, MRI	Femur	57	Chondroblastoma
Y. Tsuda, 2019	8	M	X-ray	Femur	N/A	Ewing sarcoma/osteosarcoma
Y. Tsuda, 2019	52	F	CT	Skull	60	SEF
Y. Tsuda, 2019	28	M	CT	Mandible	30	Small round cell tumor
G. Wang, 2010	57	F	X-ray, CT	Pubic	60	Not specified
C. Wei, 2022	28	F	CT	Vertebrae	80	SEF
J.B. Wojcik, 2014	51	F	X-ray, CT, MRI	Femur	96	Not specified
J.J. Xu, 2016	24	M	CT, MRI	Skull	50	Invasive meningioma
A. Popli, 2017	77	F	X-ray, CT	Coccyx	89	Chordoma/sarcoma
C. Laliberte, 2018	55	F	NM^a^	Mandible	49	Desmoplastic fibroma
S. Amer, 2024	52	M	X-ray	Mandible	19	Granuloma

M, Male; F, Female, SEF, sclerosing epithelioid fibrosarcoma.

^a^ Not mentioned. The imaging modality is not mentioned, but radiologic findings are described.

### Radiologic features

X-ray imaging findings were documented in 16 of the 26 included cases, while CT scans were reported in 13 cases. The most common X-ray and CT scan results were lysis and destruction in 23 instances (88.46%), 18 well-defined margins in 22 cases (81.81%), soft tissue involvement in 14 cases (53.84%), and sclerotic rims in 8 cases (30.76%). Other radiologic findings are shown in Fig. 2. Furthermore, eight studies reported MRI results. Four articles presented MRI T1 and T2 intensity results individually. T1 showed hypointense and isointense signals in all descriptions; T2 showed mixed hypointense and hyperintense signals in three cases, as well as a purely hypointense signal in one case. Two instances were found to have solely bone marrow edema on MRI. In one investigation, PET scan results indicated decreased activity and rim-like enhancement (reactive change).

**Figure 2.**
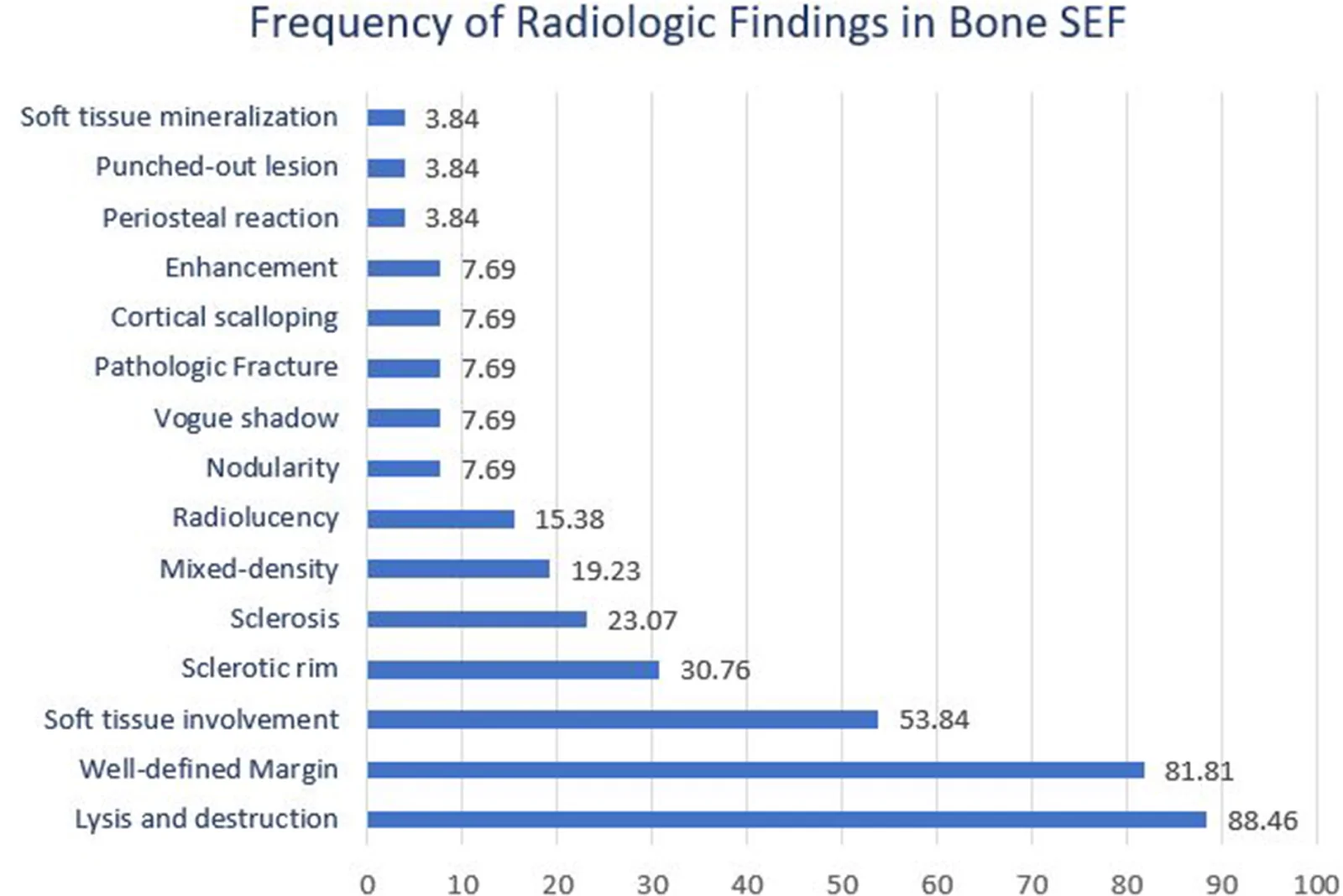
Frequency of radiologic findings of sclerosing epithelioid fibrosarcoma (SEF) of bone on computed tomography scans and X-rays.

### Radiologic findings by bone type

In terms of anatomical distribution, tumors involved irregular bones in 12 cases (46.15%), long bones in 10 cases (38.46%), and flat bones in 4 cases (15.38%). In flat bones, the most common radiologic findings were lytic lesions and well-defined margins, both present in 100% of cases, followed by soft tissue involvement in 75% of cases. In irregular bones, well-defined margins (81.8%), lysis (75%), and soft tissue involvement (58.3%) were the most frequently reported features. For long bones, lysis was observed in all cases (100%), followed by well-defined margins (75%) and a sclerotic rim in 60% of cases. The most common findings across the different bone types are summarized in Table 2.

**Table 2:** Computed tomography scan and X-ray findings in different bone subgroups.

Common radiologic features	Flat bones (*n* = 4)	Irregular bones (*n* = 12)	Long bones (*n* = 10)
Lysis, destruction, and erosion	4 (100)	9 (75)	10 (100)
Well-defined margin	3 (100)	9 (81.8)	6 (75)
Soft tissue involvement	3 (75)	7 (58.33)	4 (40)
Sclerotic rim	1 (25)	1 (8.33)	6 (60)
Intralesional classification/ sclerosis	0 (0)	3 (25)	3 (30)
CT Mixed-density	0 (0)	3 (25)	2 (20)
Maximum diameter	51.25 ± 19.31	52 ± 21.41	51.25 ± 20.24

Variables are presented as frequency (percentage).

## Discussion

The nonspecific clinical signs and symptoms, together with inconsistencies in the reported imaging characteristics of SEF of bone, have contributed to delayed or missed diagnoses and poorer outcomes for this aggressive tumor^[^6,30^]^. This systematic review delineates the distinctive radiologic characteristics of bone SEF with a categorized approach based on bone types to improve detection and interpretation across imaging modalities and to guide clinicians toward more accurate decision-making.

This investigation revealed that lytic lesions with well-defined margins, often accompanied by soft-tissue involvement, were the predominant radiologic findings. Although these findings on X-ray and CT scans may not be specific, primary SEF of the bone should be included in the differential diagnosis, particularly when multiple features are present in common sites such as the mandible or femur. MRI, however, demonstrated greater reproducibility and consistency in the diagnosis of SEF, especially through the signal characteristics on T1- and T2-weighted images^[^3,14,29^]^. Correct initial diagnosis of SEF was more frequently reported in studies that performed MRI, highlighting the potential impact of MRI findings on diagnostic accuracy and clinical practice. Of the seven studies that conducted MRI and reported an initial diagnosis, four (57.1%) correctly identified SEF of bone based on clinical and imaging findings, compared with only three of the 14 studies (21.4%) that did not use MRI.

Among the various anatomical sites, the mandible was the most commonly affected bone, with 7 cases (26.9%), followed by the femur with 5 cases (19.2%). Irregular bones (46.1%) and long bones (38.4%) were the predominant sites of involvement. Notably, there were no reported cases of SEF originating in short bones, such as those in the wrist and ankle. Radiologic findings by bone type indicated that the presence of a sclerotic rim was markedly more prevalent in lesions of long bones (60%) than in irregular and flat bones, where it was observed in 8.3% and 25% of cases, respectively.

SEF is frequently misdiagnosed as LGFMS due to overlapping clinical features^[^26,30,31^]^. Furthermore, hybrid lesions exhibiting characteristics of both tumor types have been described, suggesting a potential progression from LGFMS to SEF[20]. Despite these similarities, LGFMS is generally associated with a lower risk of recurrence and mortality, whereas SEF is characterized by a more aggressive clinical course. According to two previous reviews summarizing imaging features, LGFMS often presents with CT attenuation that is hypodense relative to muscle and a gyriform signal intensity with contrast enhancement on MRI^[^32,33^]^. While contrast enhancement is also common in SEF, other MRI characteristics, such as hypo- and isointense signals on T1-weighted images and irregular hypointense areas within the mass on T2-weighted images, may aid in differentiating the two entities[34].

A diagnosis of SEF often necessitates invasive treatment. Because SEF is frequently diagnosed at a later stage and tends to be more aggressive, lesions are typically larger and more likely to have metastasized at the time of diagnosis than those associated with LGFMS. Consequently, neoadjuvant therapy, external-beam radiotherapy, or chemoradiation administered before or during amputation or surgical excision is more commonly used in patients with SEF[31]. However, earlier and more timely diagnosis of SEF may reduce the need for such invasive treatments, although this possibility requires further investigation.

This review is limited by the rarity of bone SEF, which significantly restricts the available data and results in a lack of prospective studies, thereby limiting the generalizability of the radiological findings. The analysis relies solely on the radiological descriptions provided in the included studies, without an independent imaging assessment. Additionally, since most studies did not report X-ray and CT findings separately, a distinct analysis of these modalities was not feasible. Despite these limitations, this review constitutes the most comprehensive systematic effort to extract and categorize imaging features of bone SEF. It supports radiologists and clinicians in diagnosing this rare tumor, while also providing a valuable foundation for future research and for the development of AI-assisted models, including large language models such as ChatGPT, within emerging clinical decision support systems[35].

## Conclusion

This systematic review sheds light on the diverse imaging characteristics of bone SEF across various bone types. Although SEF often appears deceptively benign on imaging, clinicians should remain vigilant for lytic lesions, well-defined margins, subtle soft tissue extension, or sclerotic borders. When these features raise suspicion, prioritizing MRI and early biopsy can help ensure timely and accurate diagnosis and management of this aggressive sarcoma.

## Data Availability

The extracted data from the included articles are available from the corresponding author upon reasonable request.

## Research registration unique identifying number (UIN)

CRD420251005117.

## Transparency in the reporting of artificial intelligence

No AI tools were used in research or manuscript development.
